# Risk of early stroke recurrence and procedural stroke or death in carotid near occlusion patients scheduled for carotid endarterectomy

**DOI:** 10.1007/s00415-026-14198-x

**Published:** 2026-10-16

**Authors:** Henrietta Törmänen, Suvi Koskinen, Krista Nuotio, Riikka Tulamo, Pirkka Vikatmaa, Lauri Soinne, Perttu J. Lindsberg, Petra Ijäs

**Affiliations:** 1 Department of Neurology, Helsinki University Hospital, Biomedicum 1, Haartmaninkatu 8, 00029 Helsinki, Finland; 2 Clinical Neurosciences, Clinicum, University of Helsinki, Helsinki, Finland; 3 Department of Radiology, Hospital District of Helsinki and Uusimaa Medical Imaging Center, University of Helsinki and Helsinki University Hospital, Helsinki, Finland; 4 Department of Vascular Surgery, University of Helsinki and Helsinki University Hospital, Helsinki, Finland

**Keywords:** Carotid near occlusion, Carotid endarterectomy, Hyperperfusion syndrome

## Abstract

**Background:**

Carotid near occlusion (NO) refers to distal internal carotid artery (ICA) narrowing caused by proximal carotid stenosis (CS). Stroke risk and optimal management of NO remain controversial. Our aim was to determine the prevalence of NO severities, rates of preoperative ischemic cerebrovascular events (iCVEs), 30-day procedural stroke and death, and hyperperfusion syndrome in patients with NO in a prospective CS cohort undergoing carotid endarterectomy (CEA).

**Methods:**

We reanalyzed posthoc CTAs from 443 symptomatic and asymptomatic CS patients. Subtle NO (s-NO) was defined as a 1.0-mm difference in distal luminal diameter between ipsilateral and contralateral ICA. NO with full collapse (fc-NO) was defined as ≤2.0 mm and/or an ipsilateral-to-contralateral ICA diameter ratio ≤0.42. Conventional stenosis was defined as CS >50% using NASCET criteria. Rates of preoperative iCVEs and 30-day procedural stroke and death were compared between s-NO, fc-NO, and conventional stenosis.

**Results:**

NO occurred in 143 (32%) patients: 124 (87%) s-NO and 19 (13%) fc-NO. NO was not significantly associated with increased preoperative iCVEs (7.6% vs 13%, OR 1.82, 95%Cl 0.96–3.47) or procedural stroke or death (5.6% vs 3.3%, OR 1.73, 95%Cl 0.67–4.50) compared with conventional stenosis. After CEA, hyperperfusion syndrome was frequent in NO patients (7.0% vs 2.7%, OR 2.8, 95% CI1.1–7.1). One patient developed fatal hyperperfusion-related intracranial hemorrhage.

**Conclusion:**

CTA-detected NO is common, particularly in subtle forms, and important for stenosis assessment and management. Unlike earlier studies, NO was not associated with increased preoperative or procedural stroke or death, but hyperperfusion syndrome after CEA was more frequent.

**Supplementary Information:**

The online version contains supplementary material available at https://doi.org/10.1007/s00415-026-14198-x.

## Introduction

Carotid near occlusion (NO) refers to severe stenosis of the internal carotid artery (ICA) leading to a reduction in the distal diameter of the vessel. It reflects the inability to sustain adequate blood flow past a critical narrowing of the carotid stenosis [1, 2]. Unlike conventional stenosis, the severity of NO cannot be reliably expressed as a percentage reduction in luminal diameter, which complicates both diagnosis and clinical decision-making.

The clinical significance and management of NO is controversial [2, 3]. In the landmark NASCET and the European Carotid Surgery Trial (ECST) trials, only 262 (4.4%) patients with NO were included, and the observed lower benefit of CEA compared with conventional stenosis was driven by a low risk of recurrent stroke in the medically treated group rather than by increased procedural risks associated with surgery [3]. Since then, the benefit of CEA among symptomatic patients with NO has remained controversial. Furthermore, the use of digital subtraction angiography (DSA) in these early trials likely underestimated the prevalence of subtle NO, capturing primarily those with full collapse [4].

Advances in imaging, particularly computed tomography angiography (CTA), have refined the recognition of NO by integrating luminal measurements with hemodynamic features such as delayed contrast arrival and collateral circulation [3, 5]. Diagnostic thresholds including a distal ICA diameter of ≤2.0 mm or an ipsilateral-to-contralateral ICA ratio of ≤0.42 have been proposed to identify NO with full-collapse (fc-NO), which has been associated with an increased risk of early recurrent ischemic events [6]. In recent years, milder forms of collapse have been increasingly recognized and referred to as near-occlusion without full-collapse [2].

We earlier defined subtle NO (s-NO) as a side-to-side distal ICA luminal diameter difference of ≥1.0 mm, detected with CTA, but the clinical significance has not yet been delineated [5, 7].

Some reports suggest that symptomatic patients with fc-NO face a high early stroke risk when treated with best medical therapy (BMT) alone [6, 8], while others have not demonstrated this excess risk [9]. A meta-analysis by Antonopoulos et al. [10] suggests that CEA or carotid artery stenting (CAS) may reduce stroke recurrence compared with BMT, supporting their safety and effectiveness in selected patients. Nonetheless, evidence remains limited, and guideline recommendations are cautious. The 2023 European Society for Vascular Surgery (ESVS) guidelines provide only a Class IIb recommendation for revascularization in symptomatic fc-NO with recurrent events despite BMT, underscoring the persistent uncertainty surrounding optimal treatment [11].

To this end, this study aimed to investigate the prevalence of NO, including the whole range from subtle to full collapse, in patients undergoing CEA, and to explore their association with clinical characteristics and vascular risk factors, preoperative recurrent ischemic cerebrovascular events (iCVE) and procedural outcomes, including stroke, stroke-related death, all-cause death and postoperative hyperperfusion syndrome.

## Materials and methods

This article follows the STrengthening the Reporting of OBservational studies in Epidemiology (STROBE) reporting guidelines.

The Helsinki Carotid Endarterectomy Study 2 (HeCES2) prospectively recruited 500 consecutive carotid stenosis (CS) patients scheduled for CEA in Helsinki University Hospital, a single tertiary stroke center, between October 2012 and September 2015. Patients were referred for CEA from the Hospital District of Helsinki and Uusimaa with a catchment area of approximately 1.6 million.

CEA decisions were made according to our institutional guidelines, mostly aligning national guidelines based on the European Society for Vascular Surgery (ESVS) [12] and European Stroke Organization guidelines at the time [13]. Symptomatic patients were referred to by neurologist or ophthalmologist and the decision to perform CEA was made in a multidisciplinary discussion. Asymptomatic patients were operated according to the recommendations on the ESVS guidelines [12], to reduce the long-term risk of disabling or fatal strokes in patients under the age of 75 years and with a life expectancy of at least 5 years.

The study cohort was established based on the decision to perform CEA, not the operation itself; thus, patients who experienced adverse events between the operative decision and surgery were retained in the analysis.

Patients with fc-NO who experienced recurrent symptoms despite BMT or presented multiple stenoses and symptoms suspected of being hemodynamic in origin, were scheduled for CEA after multidisciplinary team evaluation. Patients with s-NO, who did not meet the NASCET-defined 'string sign' criteria, were considered for CEA based on general guidelines. During the decision-making phase, the NASCET definition of the "string sign" was applied to NO patients.

**Fig. 1:**
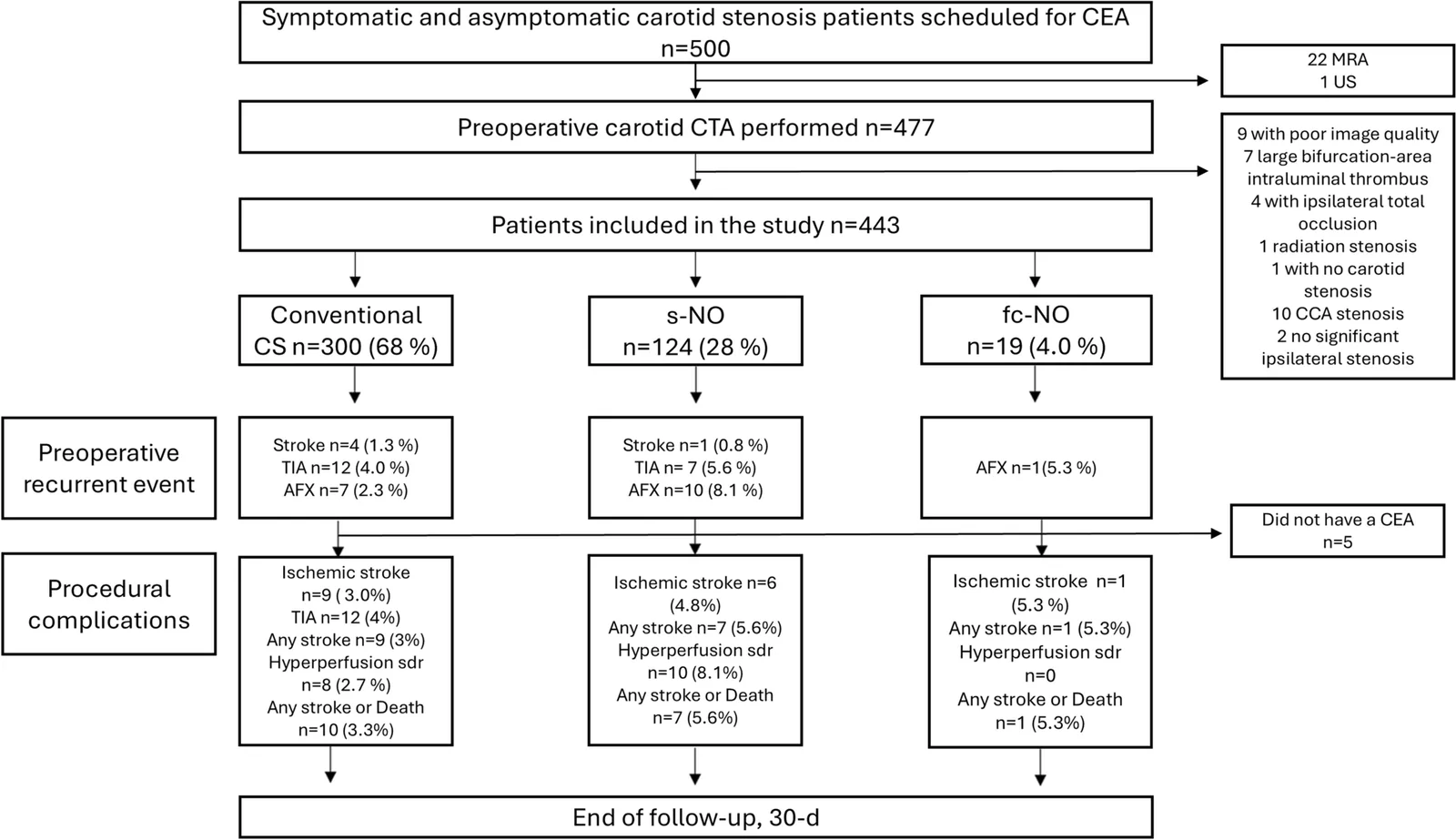
Flowchart of the study: symptomatic and asymptomatic carotid stenosis (CS) patients treated with carotid endarterectomy (CEA) and included in Helsinki Carotid Endarterectomy 2 study with carotid computed tomography angiography (CTA), magnetic resonance angiography (MRA) or ultrasound (US) available. *CCA* common carotid artery; *s-NO* subtle near-occlusion; *fc- NO* near-occlusion with full collapse; *TIA* transient ischemic attack; *AFX* amaurosis fugax

The index symptom was defined as the presenting symptom which led the patient to seek medical assistance. Based on clinical and imaging data, an experienced stroke neurologist redefined the stenosis as symptomatic or asymptomatic. Patients were classified as symptomatic if they had suffered an ipsilateral amaurosis fugax (AFX), transient ischemic attack (TIA) or ischemic stroke within 180 days prior to CEA [14]. If a clear distinction could not be made, for example in case of several potential etiologies for ischemic stroke or ambiguous symptoms in a suspicion of TIA, the CS was classified as “suspect”. If the patient had concurrent symptoms of different severity, symptoms were registered according to the most severe symptom; for example, if the patient had suffered a hemispheric TIA and an AFX, hemispheric TIA was registered as the index symptom. Symptom distribution is available in Fig. 1.

A recurrent event refers to an ischemic cerebrovascular event in the territory of the previously diagnosed CS, that occurred after the decision to perform CEA was made.

Procedural stroke was defined as a new neurological deficit lasting longer than 24 h within 30 days of CEA. An ischemic cerebrovascular event (iCVE) was defined as procedural stroke or TIA, or transient or persistent worsening of symptoms without evidence of a new ischemic lesion in head CT and not caused by procedural hypoperfusion. If a distinction could not be made, the occurring symptoms were classified as TIA/worsening of symptoms.

Hyperperfusion syndrome was diagnosed when typical symptoms (hypertension, headache, confusion, focal neurological deficits, and/or seizures) occurred in association with elevated flow velocity in the ICA territory ipsilateral to CEA, compared to the contralateral ICA, as measured by transcranial doppler (TCD) in the middle cerebral artery. We did not apply a definite cut-off value since it has been shown that hyperperfusion can occur in the presence of moderate relative hyperperfusion of the ipsilateral hemisphere [15].

Preoperative CTAs were performed either in the referring unit or in our centre. Two experienced neuroradiologists and a radiology resident, trained to analyze carotid CTAs, conducted all reanalyses and measurements, as described in detail elsewhere [5]. NASCET-criteria were used for the measurement of the degree of stenosis [16].

Of the whole study cohort, 477 patients underwent pre-operative carotid CTA, 22 magnetic resonance angiographies, and one patient with contraindications for both underwent duplex sonography only. For diagnostic consistency, we included only CTA examinations in this analysis. Of the 477 CTAs, we excluded 34 for reasons defined in Fig. 1, leaving 443 CTAs available for analysis.

The cutoff for a significant CS was defined as 50% or over. We recorded CS as moderate (50–69%) or severe (70–99%) according to NASCET grading [16], and bilateral disease as the presence of bilateral significant stenosis.

**Fig. 2:**
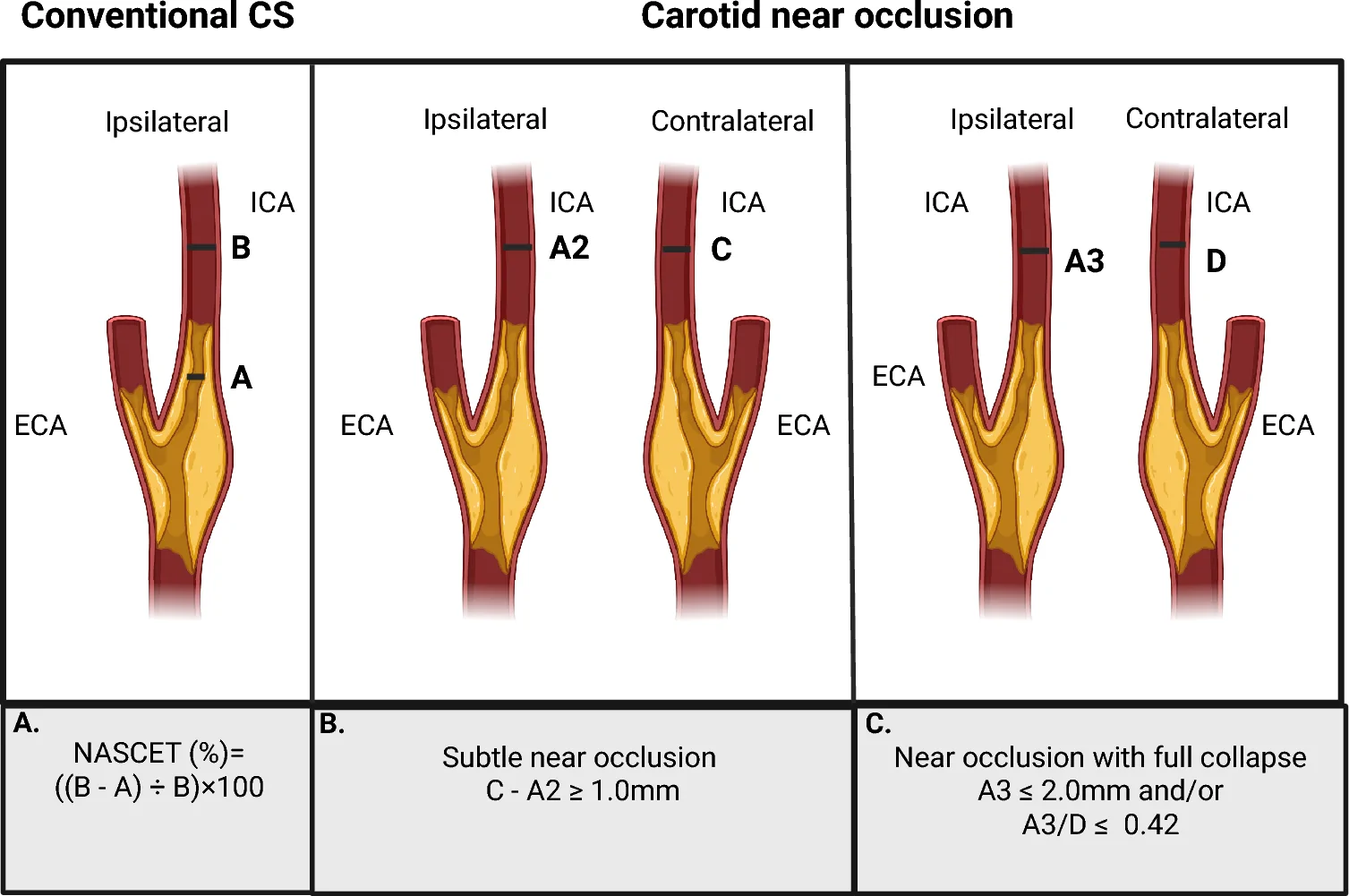
Definitions of conventional carotid stenosis, subtle near-occlusion (s-NO), and near-occlusion with full collapse (fc-NO). **A** Conventional carotid stenosis is defined by NASCET as percentage diameter reduction at the stenosis compared with the distal normal ICA. **B** s-NO is a ≥1.0 mm side-to-side difference in distal ICA diameter without full collapse. **C** fc-NO is defined as distal ICA diameter ≤2.0 mm and/or an ipsilateral-to-contralateral ICA ratio ≤0.42

*S-NO* was defined as a ≥ 1.0-mm difference in distal luminal diameter (LD) between ipsilateral and contralateral ICA [5, 7]. If the contralateral stenosis was severe (70–99%), there was total occlusion, or NO was suspected, this side-to-side criterion could not be used but visual neuroradiological assessment was performed. There were 13 such cases in the current study. *Fc-NO* was defined as an ipsilateral distal ICA diameter ≤ 2.0 mm and/or an ipsilateral-to-contralateral ICA diameter ratio ≤ 0.42 [8]. Definitions are provided in Fig. 2. By definition, all patients with fc-NO also fulfil the criteria for s-NO. However, in the current study, they were kept in separate groups so that differences between the subgroups could be compared.

The study protocol was approved by the Helsinki and Uusimaa Medical Ethics Committee. The study was carried out in accordance with the ethical principles of the Helsinki Declaration. All patients signed an informed consent.

Demographic data of the patients are presented as mean and standard deviation for variables with a normal distribution, median and interquartile range for non-normal or ordinal variables and percentages for nominal variables. For dichotomous variables, the Chi-square or Fisher’s exact test was applied to test for differences between groups. Student’s T-test was used for continuous variables with a normal distribution, while Mann–Whitney U-test was employed for comparisons of continuous non-normally distributed variables. Age-adjusted odds ratios (OR) were calculated by logistic regression. Because of the exploratory nature of the analysis, the results were not corrected for multiple comparison. For statistical analyses, we utilized IBM SPSS Statistics version 22.

## Results

### Description of the study cohort

In total, 443 CS patients with available carotid CTA were included in these analyses (Table 1, Fig. 1). The mean age of patients was 70 (± 8.6), and 68% were male. The most prevalent index symptom was hemispheric stroke (27%); the remaining had AFX (18%), TIA (17%), or retinal artery occlusion (RAO, 4.7%). Seven patients (1.6%) had ocular ischemic syndrome (OIS). 88 (20%) patients were asymptomatic, while in 61 (14%) symptoms were classified as ‘suspect’. Symptomatic patients underwent surgery after a median delay of 12 days from the index symptom (IQR 7–19).

**Table 1:** Baseline demographics and clinical characteristics in the carotid endarterectomy patients with ipsilateral conventional carotid stenosis (CS) or with ipsilateral near-occlusion

	All CS Patients With CTA Imaging, *n* = 443	Ipsilateral Conventional CS, *n* = 300; 68%	Ipsilateral Near-occlusion, *n* = 143; 32%	*p* value*
Female gender—%	140 (32)	97 (32)	43 (30)	.66
Mean age ± SD—years	70 ± 8.6	70 ± 8.4	69 ± 9.1	.39
*Risk factors and comorbidities—%*				
Smoking	160 (36)	100 (33)	60 (42)	.09
Heavy alcohol consumption	49 (11)	27 (9.0)	22 (15)	.05
Hypercholesterolemia	406 (92)	277 (92)	129 (90)	.47
Arterial hypertension	358 (81)	246 (82)	112 (78)	.37
DM	145 (33)	91 (30)	54 (38)	.13
Metabolic syndrome	179 (40)	115 (38)	64 (45)	.22
Coronary artery disease	161 (36)	117 (39)	44 (31)	.11
Peripheral arterial disease	76 (17)	54 (18)	22 (15)	.59
Atrial fibrillation	71 (16)	54 (18)	17 (12)	.13
Chronic kidney failure	63 (14)	43 (14)	20 (14)	1.00
Previous carotid territory symptom	81 (18)	60 (20)	21 (15)	.19
*Index symptom—%*				
Ischemic stroke	119 (27)	81 (27)	38 (27)	1.0
RAO	21 (4.7)	15 (5.0)	6 (4.2)	.81
TIA	75 (17)	48 (16)	27 (19)	.50
AFX	79 (18)	42 (14)	37 (26)	<.01
OIS	7 (1.6)	5 (1.7)	2 (1.4)	1.00
Asymptomatic	88 (20)	69 (23)	19 (13)	.02
Suspect	61 (14)	45 (15)	16 (11)	.31
*Recanalization therapy and presentation—%*				
IVT	19 (4.3)	13 (4.3)	6 (4.2)	1.00
EVT	0	0	0	
*Medications—%*				
SAPT	384 (87)	260 (68)	124 (32)	1.00
DAPT	19 (4.3)	13 (4.3)	6 (4.2)	1.00
LMWH	199 (45)	116 (39)	83 (58)	<.001
DM medication	135 (31)	86 (29)	49 (34)	.27
Antihypertensive medication	363 (82)	253 (84)	110 (77)	.07
Lipid-lowering medication	318 (72)	223 (74)	95 (66)	.09
Radiology				
contralateral moderate stenosis	32 (7.2)	21 (7.0)	11 (7.7	.85
contralateral severe stenosis	33 (7.4)	22 (7.3)	11 (7.7)	1.0
contralateral near-occlusion	14 (3.2)	12 (4.0)	2 (1.4)	.24
contralateral occlusion	27 (6.1)	27 (9.0)	0	<.001
Time to CEA, median (IQR)	12 (7–19)	12 (7–19)	11 (7–21)	0.46

Data are presented as n (%) and mean ± standard deviation (SD). Conventional stenosis includes moderate or severe stenosis using NASCET criteria without near-occlusion *CTA* computed tomography; *DM* diabetes mellitus; *RAO* retinal artery occlusion; *TIA* transient ischemic attack; *AFX* amaurosis fugax; *IVT* intravenous thrombolysis, *EVT* endovascular treatment; *SAPT* single antiplatelet therapy; *DAPT* dual antiplatelet therapy; *LMWH* low molecular weight heparin

^*^p <.05 is considered statistically significant

### Prevalence of NO and association with demographics and clinical characteristics

Altogether 143 ipsilateral NOs (32%) in preoperative CTA were detected, of which 124 were subtle NO (s-NO, 87%) and 19 showed full collapse (fc-NO, 13%) (Fig. 1). All cases of NO had been classified as high-grade CS. There were no differences in vascular risk factors or comorbidities between patients with NO or conventional stenosis (Table 1). AFX as an index symptom was more common in NO patients than in patients with conventional stenosis (26% vs. 14%, *p* = 0.003) and it tended to be more commonly repetitive (*p* = 0.058), with a median of 3 (range 1–50) attacks per patient before hospital admission and the decision to perform CEA.

Demographic and clinical characteristics of patients with NO divided into s-NO and fc-NO are shown in supplemental Table 1. In exploratory subgroup analyses that were not adjusted for multiple comparisons, fc-NO patients more often reported earlier ipsilateral carotid symptoms prior to the index event (*p* = 0.037), but less frequently had hypertension (*p* = 0.033), diabetes (*p* = 0.042), and metabolic syndrome (*p* = 0.006) compared to patients with s-NO.

When compared to patients with conventional stenosis, fc-NO patients less frequently had hypertension (58% vs. 82%, *p* = 0.017), used antihypertensive medication (58% vs. 84%, *p* = 0.008), and had known coronary artery disease (11% vs. 39%, *p* = 0.013) (Table 2). Effect estimates and p-values are provided for descriptive purposes. The s-NO group contained fewer asymptomatic patients (14% vs. 23%, p= 0.034), but more patients with AFX (27% vs. 14%, *p* = 0.003) than the conventional CS group (Table 3). Metabolic syndrome (49% vs. 38%, *p* = 0.040) and diabetes (41% vs. 30%, *p* = 0.041) were more common in the s-NO group compared to the conventional CS group.

**Table 2:** Baseline demographics, clinical characteristics, preoperative and procedural 30-day outcomes in the carotid endarterectomy patients with ipsilateral conventional carotid stenosis (CS) or with ipsilateral near-occlusion (NO) with full collapse

	All CS Patients, *n* = 319	Conventional Stenosis, *n* = 300, 94%	No With Full Collapse, *n* = 19, 6%	*p* value*	Age-adjusted OR (95% CI) *
Female gender—%	103 (32)	97 (32)	6 (32)	1.0	1.11 (0.10–13.0)
Mean age ± SD—years	70 ± 8.5	70 ± 8.4	65 ± 11	.07	
*Risk factors and comorbidities*					
Smoking	109 (34)	100 (33)	9 (47)	.22	1.02 (0.35–2.96)
Heavy alcohol consumption	31 (9.7)	27 (9.0)	4 (21)	.10	2.12 (0.62–7.24)
Hypercholesterolemia	293 (92)	277 (92)	16 (84)	.19	0.46 (0.11–1.95)
Arterial hypertension	257 (81)	246 (82)	11 (58)	.02	0.31 (0.12–0.82) *
DM	94 (30)	91 (30)	3 (16)	.21	0.45 (0.13–1.61)
Metabolic syndrome	118 (37)	115 (38)	3 (16)	.05	0.32 (0.09–1.13)
Coronary artery disease	119 (37)	117 (39)	2 (11)	.01	0.27 (0.06–1.22)
Peripheral arterial disease	57 (18)	54 (18)	3 (16)	1.00	0.92 (0.25–3.37)
Atrial fibrillation	55 (17)	54 (18)	1 (5.3)	.22	0.30 (0.04–2.60)
Chronic kidney failure	44 (14)	43 (14)	1 (5.3)	.49	1.00 (0.10–9.20)
Previous carotid territory symptom	66 (21)	60 (20)	6 (32)	.24	1.81 (0.65–5.02)
*Index symptom*					
Ischemic stroke	89 (28)	81 (27)	8 (42)	.19	2.18 (0.83–5.72)
RAO	15 (4.7)	15 (5.0)	0	1.0	-
TIA	51 (16)	48 (16)	3 (16)	1.0	1.14 (0.31–4.25)
AFX	46 (14)	42 (14)	4 (21)	.50	1.35 (0.40–4.53)
Asymptomatic	71 (22)	69 (23)	2 (11)	.27	0.39 (0.09–1.75)
Suspect	47 (15)	45 (15)	2 (11)	1.0	0.67 (0.15–2.96)
*Recanalization therapy*					
IVT	13 (4.1)	13 (4.3)	0	1.0	-
EVT	0	0	0	-	-
*Medication use preoperatively*					
SAPT	276 (87)	260 (86)	16 (84)	.73	0.82 (0.23–2.94)
DAPT	14 (4.4)	13 (4.3)	1 (5.3)	.59	1.83 (0.15–22.3)
LMWH	162 (51)	149 (50)	13 (68)	.16	1.64 (0.64–4.23)
DM medication	88 (28)	86 (29)	2 (11)	.11	0.30 (0.07–1.33)
Antihypertensive medication	264 (83)	253 (84)	11 (58)	.01	0.26 (0.10–0.70) *
Lipid-lowering medication	235 (74)	223 (74)	12 (63)	.29	0.78 (0.27–2.30)
*Pre- and perioperative outcomes*					
Recurrent iCVE	24 (7.5)	23 (7.7)	1 (5.3)	1.00	0.58 (0.07–4.62)
Recurrent stroke	4 (1.3)	4 (1.3)	0	1.00	0.99 (0.97–1.00)
Recurrent TIA	12 (3.8)	12 (4.0)	0	1.00	0.99 (0.97–1.00)
Recurrent AFX	8 (2.5)	7 (2.3)	1 (5.3)	.39	1.49 (0.16–14.2)
*Procedural complications*					
Procedural *iCVE*	22 (6.9)	21 (7.0)	1 (5.3)	1.00	0.87 (0.11–6.96)
Procedural stroke	10 (3.1)	9 (3.0)	1 (5.3)	.46	1.78 (0.21–15.3)
*Procedural* TIA/symptom worsening	12 (83.8)	13 (4.0)	0	1.0	
Procedural AMI/AP	8 (2.5)	7 (2.3)	1 (5.3)	.39	3.14 (0.35–28.2)
Bleeding complication	33 (10)	32 (11)	1 (5.3)	.71	0.51 (0.07–3.95)
Hyperperfusion syndrome	8 (2.5)	8 (2.7)	0	1.00	0.97 (0.96–0.99)
Any stroke within 30 d	10 (3.1)	9 (3.0)	1 (5.3)	.46	1.78 (0.21–15.3)
Any stroke or death within 30 d	11 (3.4)	10 (3.3)	1 (5.3)	.50	1.79 (0.21–15.1)

Data are presented as n (%), mean ± standard deviation, or median (range). *OR* odds ratio; *CI* confidence interval; *DM* diabetes mellitus; *RAO* retinal artery occlusion; *TIA* transient ischemic attack; *AFX* amaurosis fugax; *IVT* intravenous thrombolysis; *EVT* endovascular treatment; *AMI* acute myocardial infarction; *AP* angina pectoris; *iCVE* ischemic cerebrovascular event; *SAPT* single antiplatelet therapy; *DAPT* dual antiplatelet therapy; *LMWH* low molecular weight heparin

^*^The p value and OR is calculated between the conventional CS and fc-NO groups. *p* <.05 is considered statistically significant

**Table 3:** Baseline demographics, clinical characteristics, preoperative and procedural 30-day outcomes in the carotid endarterectomy patients with ipsilateral conventional carotid stenosis (CS) or subtle near occlusion (NO)

	All CS Patients With CTA *n* = 424	Conventional CS *n* = 300; 71%	Subtle No *n* = 124; 29%	*p* value*	Age-adjusted OR (95% CI) *
Female gender	134 (32)	97 (32)	37 (30)	.65	3.09 (0.84–11.4)
Mean age ± SD—years	70 ± 8.4	70 ± 8.4	70 ± 8.6	.91	
*Risk factors and comorbidities*					
Smoking	151 (36)	100 (33)	51 (41)	.15	1.46 (0.90–2.40)
Heavy alcohol consumption	45 (11)	27 (9.0)	18 (15)	.12	1.80 (0.90–3.43)
Hypercholesterolemia	390 (92)	277 (92)	113 (91)	.70	0.90 (0.37–1.82)
Arterial hypertension	347 (82)	246 (82)	101 (82)	.89	1.61 (0.61–4.30)
DM	142 (34)	91 (30)	51 (41)	.04	1.41 (0.84–2.40)
Metabolic syndrome	176 (42)	115 (38)	61 (49)	.04	1.40 (0.82–2.32)
Coronary artery disease	159 (38)	117 (39)	42 (34)	.38	0.82 (0.52–1.30)
Peripheral arterial disease	73 (17)	54 (18)	19 (15)	.57	0.73 (0.41–1.32)
Atrial fibrillation	70 (17)	54 (18)	16 (13)	.25	0.69 (0.37–1.30)
Chronic kidney failure	62 (15)	43 (14)	19 (15)	.77	1.23 (0.70–2.30)
Previous carotid territory symptom	75 (18)	60 (20)	15 (12)	.07	0.60 (0.30–1.03)
*Index symptom*					
Ischemic stroke	111 (26)	81 (27)	30 (24)	.63	1.36 (0.76–2.42)
RAO	21 (5.0)	15 (5.0)	6 (4.8)	1.0	1.47 (0.53–4.10)
TIA	72 (17)	48 (16)	24 (19)	.40	1.84 (0.98–3.45)
AFX	75 (18)	42 (14)	33 (27)	<.01	2.87 (1.58–5.31) *
Asymptomatic	86 (20)	69 (23)	17 (14)	.03	0.89 (0.81–0.98) *
Suspect	59 (14)	45 (15)	14 (11)	.36	0.956 (0.89–1.04)
*Recanalization therapy*					
IVT	19 (4.5)	13 (4.3)	6 (4.8)	.80	1.12 (0.42–3.02)
EVT	0	0	0	-	-
*Medication use preoperatively*					
SAPT	368 (87)	260 (87)	108 (87)	1.00	1.04 (0.56–1.93)
DAPT	18 (4.2)	13 (4.3)	5 (4.0)	1.00	0.93 (0.32–2.70)
LMWH	231 (55)	149 (50)	82 (66)	<.01	2.40 (1.56–3.71) *
DM medication	133 (31)	86 (29)	47 (38)	.07	1.52 (0.98–2.36)
Antihypertensive medication	352 (83)	253 (84)	99 (80)	.26	0.74 (0.43–1.30)
Lipid-lowering medication	306 (k72)	223 (74)	83 (67)	.12	0.70 (0.43–1.11)
*Preoperative and proceduraloutcomes*					
*Recurrent iCVE*	41 (9.7)	23 (7.7)	18 (15)	.04	2.04 (1.06–3.94) *
Recurrent stroke	5 (1.2)	4 (1.3)	1 (0.8)	1.00	0.60 (0.07–5.45)
Recurrent TIA	19 (4.5)	12 (4.0)	7 (5.6)	.45	1.43 (0.55–3.74)
Recurrent AFX	17 (4.0)	7 (2.3)	10 (8.1)	.01	3.68 (1.36–9.92) *
*Procedural complications*					
Procedural iCVE	27 (6.4)	21 (7.0)	6 (4.8)	.52	0.68 (0.27–1.72)
Procedural stroke	15 (3.5)	9 (3.0)	6 (4.8)	.39	1.65 (0.57–4.73)
Procedural TIA/ symptom worsening	12 (2.8)	12 (4.0)	0	.02	
Procedural AMI/AP	10 (2.4)	7 (2.3)	3 (2.4)	1.0	1.04 (0.26–4.10)
Bleeding complication	44 (10)	32 (11)	12 (9.7)	.86	0.90 (0.45–1.81)
Hyperperfusion syndrome	18 (4.2)	8 (2.7)	10 (8.1)	.02	3.20 (1.23–8.32) *
Any stroke within 30 d	16 (3.8)	9 (3.0)	7 (5.6)	.26	1.94 (0.71–5.33)
Any stroke or death within 30 d	17 (4.0)	10 (3.3)	7 (5.6)	.28	1.74 (0.65–4.70)

Data are presented as n (%) or mean ± standard deviation

*DM* diabetes mellitus; *RAO* retinal artery occlusion; *TIA* transient ischemic attack; *AFX* amaurosis fugax; *IVT* intravenous thrombolysis; *EVT* endovascular treatment; *AMI* acute myocardial infarction; *AP* angina pectoris; *iCVE* ischemic cerebrovascular event; *SAPT* single antiplatelet therapy; *DAPT* dual antiplatelet therapy; *LMWH* low molecular weight heparin

^*^The p value and OR is calculated between the conventional CS and s-NO groups. *p* < 0.05 is considered statistically significant

### NO and early preoperative recurrent symptoms

Of all NO patients, 19 (13%) had a recurrent iCVE whilst waiting for CEA (Table 4). There was no significant difference in recurrent iCVEs between the NO and conventional stenosis (age-adjusted OR 1.82, 95% Cl 0.96–3.47). The only recurrent stroke in NO patients occurred in a symptomatic patient with s-NO. The most common recurrent event before CEA in NO patients was AFX (age-adjusted OR 3.37, 95% confidence interval [Cl] 1.27–8.93) followed by TIA (age-adjusted OR 1.23, 95% Cl 0.47- 3.21) (Table 4).

**Table 4:** Preoperative recurrent ischemic events and procedural complications in carotid stenosis (CS) patients scheduled for carotid endarterectomy (CEA) divided by the presence of ipsilateral near-occlusion (NO) (including bothsubtle NO and NO with full-collapse)

	All CS Patients With CTA Imaging, *n* = 443	Ipsilateral Conventional CS, *n* = 300; 68%	Ipsilateral Near-occlusion, *n* = 143; 32%	Age-adjusted OR (95% CI)*
*Preoperative recurrent ischemic events*				
Any recurrent iCVE	42 (9.5)	23 (7.6)	19 (13)	1.82 (0.96–3.47)
Recurrent ischemic stroke	5 (1.1)	4 (1.3)	1 (0.7)	0.53 (0.06–4.80)
Recurrent TIA	19 (4.3)	12 (4.0)	7 (4.9)	1.23 (0.47- 3.21)
Recurrent AFX	18 (4.1)	7 (2.3)	11 (7.7)	3.37 (1.27–8.93) *
CEA performed	438 (99)	296 (99)	142 (99)	1.18 (0.76–1.84)
*Procedural complications*				
Procedural iCVE	28 (6.3)	21 (7.0)	7 (4.9)	0.70 (0.29–1.68)
Procedural ischemic stroke	16 (3.6)	9 (3.0)	7 (4.9)	1.66 (0.60–4.55)
Procedural TIA/symptom worsening	12 (2.7)	12 (4.0)	0	p=0.01
Hyperperfusion syndrome	18 (4.1)	8 (2.7)	10 (7.0)	2.78 (1.06–7.12) *
Procedural AMI/AP	11 (2.5)	7 (2.3)	4 (2.8)	1.24 (0.36–4.33)
Bleeding complication	45 (10)	32 (11)	13 (9.1)	0.85 (0.43–1.68)
Any stroke within 30d	17 (3.8)	9 (3.0)	8 (5.6)	1.91 (0.72–5.06)
Any stroke or death within 30d	18 (4.1)	10 (3.3)	8 (5.6)	1.73 (0.67–4.50)

Data are presented as n (%). *iCVE* ischemic cerebrovascular event; *CTA* computed tomography; *TIA* transient ischemic attack; *AFX* amaurosis fugax; *OR* odds ratio; *CI* confidence interval; *AMI* acute myocardial infarction; *AP* angina pectoris

^*^Statistically significant

### NO and procedural complications

There were no differences in the procedural 30-d iCVEs between patients with NO and conventional CS (7/4.9% vs. 21/7.0%, age-adjusted OR 0.70, 95% Cl 0.29–1.68)(Table 4). NO patients, all of them with s-NO, more often developed hyperperfusion syndrome after CEA (10/7.0% vs. 8/2.7%, age-adjusted OR 2.78, 95% Cl 1.06–7.12) than conventional CS patients (Table 4). One s-NO patient suffered a fatal intracranial haemorrhage due to hyperperfusion syndrome. There were no significant differences in other procedural complications between patients with s-NO and fc-NO (Supplemental Table 1).

### The effect of contralateral stenosis on preoperative and procedural complications

In a sensitivity analysis excluding patients with hemodynamically significant contralateral carotid artery disease (moderate or severe stenosis, near-occlusion, or occlusion), the direction and trends of results remained consistent with primary analyses. Compared with conventional CS patients, patients with NO had a higher rate of recurrent iCVEs (15.1% vs. 7.8%, p = 0.041), driven by amaurosis fugax events (9.2% vs. 1.4%, p < 0.001), whereas recurrent stroke rates were similar (0.8% vs. 1.8%, p = 0.66). No significant differences were observed in perioperative iCVEs (3.4% vs. 6.9%, p = 0.222) or hyperperfusion syndrome (5.0% vs. 2.3%, p = 0.21). However, the number of patients with hyperperfusion syndrome was more than twice as high in the NO group as in the conventional stenosis group.

## Discussion

In this prospective cohort study including consecutive CEAs in a large tertiary stroke center, patients with NO, compared to conventional stenosis, did not carry a higher risk of preoperative recurrent stroke, nor stroke or death within 30 days of CEA. However, patients with NO more often developed hyperperfusion syndrome after CEA, necessitating meticulous blood pressure control and prolonged hospital stay, and potentially predisposing them to procedural complications, particularly intracerebral haemorrhage, if left untreated. Furthermore, our study shows that subtle NO, detectable by CTA, is more common (29%) than in NASCET and ESCT study cohorts [17, 18], a difference likely attributable to CTA being more sensitive in diagnosing NO than unilaterally performed DSA. Despite the assumed clinical relevance of NO, considerable uncertainty regarding the optimal management of NO remains. This study contributes to the evolving understanding of NO.

Identifying NO is challenging because no diagnostic gold standard exists. Diagnosis relies on a systematic assessment of imaging features, specifically, whether the distal ICA is reduced in size and whether a proximal high-grade stenosis is the most likely cause. Although measurement-based criteria have been proposed, they remain unvalidated. Detecting full collapse, traditionally defined as a threadlike distal lumen, is also imperfect. Our study used precise, reproducible measurements to define NO. The definition of s-NO was based on distal ICA diameter variability in healthy individuals [5, 7] using a 2.5 × SD cut-off (SD 0.4 mm) to provide an objective and practical tool for clinical use. While several alternative, largely visually based definitions exist, their reproducibility is limited; nevertheless, reported prevalences are similar [19]. Hemodynamic impairment in NO is well established, but the clinical relevance of s-NO remains uncertain [20].

Earlier studies have presented contradictory findings regarding early stroke recurrence in NO patients. The CAOS (CAsi Oclusión Sintomática) study reported low stroke recurrence rates for symptomatic NO patients on BMT, with 4% at 90 days, and even 0% for patients with fc-NO, compared to 6% in NO patients without full collapse [21]. In contrast, Johansson et al. reported a 90-day recurrent ipsilateral ischemic stroke risk of 43% for patients with fc-NO and 0% for those with NO without full collapse [8]. In a subsequent study by the same group, a high risk of recurrence was still observed in NO patients, though the difference between those with and without full collapse was less pronounced (30% and 22%, respectively) [22]. In our study, patients with NO did not suffer more recurrent strokes compared to the conventional CS group (0.8% in s-NO and 0% in fc-NO). The slightly higher rate of preoperative iCVEs observed in NO was primarily attributable to amaurosis fugax, which is often recurrent in nature. However, it is worth noting that the definition of NO in our study differed from the definition used by Johansson et al. [8]; who adopted the CTA criteria developed by Bartlett et al. (2006) that are more complicated yet requiring visual assessment. Our criteria are simpler and more useful for screening s-NO.

Garcia-Pastor et al. reported high technical failure and notable periprocedural complications in NO patients, including a 6% stroke or death rate [23]. In contrast, a recent meta-analysis found much lower 30-day stroke or death rates after CEA (1.8%) and CAS (2.2%) in NO patients [24]. In our cohort, the 30-day stroke/death rate in NO was 5.6% (5.6% in s-NO; 5.3% in fc-NO). NO patients did not experience more procedural iCVEs or bleeding but had more hyperperfusion syndrome. One s-NO patient died from hyperperfusion-related ICH.

Hyperperfusion syndrome occurs in 0–3% after carotid revascularization [25]. Cay et al. reported higher rates after CAS (8.6%) and particularly in fc-NO patients (30%) [26]. Garcia-Pastor et al. found a 3% rate without differences between CAS and CEA or between NO subgroups [23]. In our study, hyperperfusion occurred in 4.1% overall and in 7.0% of NO patients (age-adjusted OR 2.78). Notably, 55% of affected patients had s-NO. Although NO increases risk, it does not fully explain all cases. The predominance of s-NO may reflect impaired cerebrovascular reserve and autoregulation, promoting post-revascularization hyperperfusion. Overall, evidence indicates elevated hyperperfusion risk in NO, underscoring the need for strict postoperative blood pressure management to prevent complications, especially ICH.

In our cohort, patients with NO often had recurrent AFX as an index symptom. We interpret this finding to result from patient selection to CEA, since patients with NO are typically managed with BMT after their first symptom in accordance with the guidelines, with invasive treatments like CEA reserved for recurrent symptoms. This creates a strong association between surgery and recurrence and may also bias the prevalence of symptoms towards AFX, which is often repetitive in nature. However, we cannot rule out a true association between NO and AFX. High-grade CS reduces arterial flow, increasing embolic risk and predisposing to ocular hypoperfusion, which can lead to AFX or OIS[27]. While larger emboli rarely enter the ophthalmic artery due to its steep angle, in addition to frank hemodynamic failure, microemboli also can cause repetitive symptoms. It is plausible that the presence of NO as an indicator of that hemodynamic insuffiency is associated with a higher incidence of repetitive AFX. In our study, AFX was more common in NO than in conventional CS, even though some patients with AFX were likely not selected for surgery. In contrast, no increase was observed in the rates of stroke or hemispheric TIA, and these event rates were lower in both subtle and full-collapse near-occlusion than in conventional stenosis.

The prevalence of NO among patients with symptomatic CS ≥50% is consistent in studies with robust methodologies which report a range of 27–34%[19]. The challenge lies in accurately identifying NO, as no single diagnostic modality as a gold standard. DSA, CTA, and carotid duplex ultrasonography each have inherent limitations and risks [28, 29]. The diagnosis is established through a systematic assessment of imaging features, specifically by determining whether the distal ICA appears reduced in calibre and whether a proximal high-grade stenosis is the most plausible explanation[2, 30]. Although measurement-based diagnostic criteria have been proposed, they have not been successfully validated[28, 31]. Furthermore, detecting full collapse, traditionally defined by the presence of a threadlike arterial lumen distal to the stenosis, may not be entirely reliable. In the present study, NO was defined using precise measurement-based criteria, facilitating reproducibility in future research. Accurate recognizion of NO is essential when quantifying CS.

These findings highlight important considerations for managing NO patients undergoing CEA. Their higher risk of hyperperfusion syndrome increases the likelihood of procedural complications, making strict perioperative blood pressure control essential to prevent ICH and related events. Because optimal treatment strategies remain uncertain, further observational studies comparing NO patients with and without collapse are needed to better guide clinical decisions.

## Limitations

Our study has several limitations. Technical exclusions were required to ensure reliable CTA data. The NO definition, based on normal ICA variation, may be imperfect, as NO occurs only in severe stenosis and cannot be applied with contralateral ICA occlusion. Selection bias is possible because all patients were scheduled for CEA and no BMT-only group was available. Residual lumen measurements in very tight stenoses may be unreliable due to calcification and beam-hardening, so distal ICA measurements were used when feasible and the 1.0-mm cut-off applied only with significant bifurcation stenosis and expert neuroradiological review. The small fc-NO subgroup, reflecting limited intervention use, may have reduced power to detect small risk differences. Patients with isolated AFX, minor recurrence risk, or disabling strokes were likely not considered for surgery. Finally, the HeCES2 cohort includes patients treated over a decade ago, during which imaging, medical therapy, and perioperative practices have evolved, although core indications and surgical techniques have remained stable.

## Supplementary Information

Below is the link to the electronic supplementary material.

Supplementary file1 (DOCX 26 KB)

## Data Availability

The data that support the fi ndings of this study are available on reasonable request from the corresponding author. The data are not publicly available due to privacy or ethical restrictions.
