# Supplementary material for: Risk of early stroke recurrence and procedural stroke or death in carotid near occlusion patients scheduled for carotid endarterectomy
Source: J Neurol. 2026 Oct 16;273(11):667. doi: 10.1007/s00415-026-14198-x (PMC13675380; doi:10.1007/s00415-026-14198-x)
Supplement: Supplementary file 1 [file 415_2026_14198_MOESM1_ESM.docx]

**Risk of early stroke recurrence and procedural stroke or death in carotid near occlusion**

**^1,2^Henrietta Törmänen, ^2,3^Suvi Koskinen, ^1,2^Krista Nuotio, ^4^Riikka Tulamo, ^4^Pirkka Vikatmaa, ^2^Lauri Soinne, ^1,2^Perttu J. Lindsberg, ^1,2^Petra Ijäs.**

**Supplemental methods**

**Definition of cerebrovascular symptoms in carotid stenosis**

Index symptom was defined as the presenting symptom which led the patient to seek medical help. Based on clinical and imaging data, experienced stroke neurologist redefined the stenosis as symptomatic or asymptomatic. Patient was classified as symptomatic if she/he had suffered an ipsilateral amaurosis fugax (AFX), transient ischaemic attack (TIA) or ischemic stroke within 180 days prior to CEA. If the symptom seemed to be caused by hypoperfusion, the stenosis was classified as asymptomatic. If a clear distinction could not be made, for example in case of several possible etiologies for ischemic stroke or ambiguous symptoms in a suspicion of TIA, the CS was classified as “suspect”. Index symptoms were registered according to the most severe symptom. A consensus panel of stroke neurologists reviewed cases with uncertainty regarding stenosis symptom status, focusing on defining the etiology of symptoms, as detailed in a previous article^1^. Symptom distribution is available in Figure 1.

The number of attacks is the number of cerebrovascular symptoms associated with ipsilateral carotid stenosis, which the patient had experienced before seeking medical assistance, and before the decision to perform CEA was made.

**Other definitions**

Current smokers and ex-smokers who had stopped less than 12 months previously were classified as smokers.

Heavy alcohol consumption: doses over 12-16 for females and 23-24 for males per weeks (1 dose = 12 g pure ethanol).

Patient was defined as having metabolic syndrome if he/she had ≥3 of the following: (1) triglycerides ≥1.7 mmol/L, (2) HDL cholesterol <1.0 mmol/L in men and <1.3 mmol/L in women, (3) diagnosis of hypertension or blood pressure medication, (4) fasting glucose ≥5.6 mmol/L or diagnosis or medication of diabetes mellitus.

Patients marked as being on lipid-lowering medication had lipid-lowering medication at least 2 weeks preoperatively.

**Carotid endarterectomies**

In 5 of the originally included patients, the decision to perform CEA was withdrawn (Figure 1) for following reasons: 1 patient had no proximal ICA stenosis but prestenotic collapse and stenosis at ICA siphon, 1 was frail with asymptomatic ICA stenosis, 1 declined CEA, 1 had a major stroke whilst waiting for CEA, and 1 had poor recovery from the index stroke why CEA was cancelled. Four of these patients had conventional CS and one s-NO. Regarding the index symptom, 1 had a stroke, 1 had AFX and 3 were asymptomatic or the etiology of their cerebrovascular symptoms was unclear. All patients with fc-NO were operated on as planned.

All other patients had patch angioplasty, a polyester-urethane patch under either general (GA) or locoregional anaesthesia (LA). The preferred monitoring method was LA combined with transcranial doppler (TCD) and near-infrared spectroscopy (NIRS) but was not always feasible due to non-compliance to LA and non-existing TCD window in some patients. Shunting was performed selectively based on the surgeon’s evaluation of the findings in TCD and/or NIRS or symptoms in the awake patient. Routine completion control was achieved using transit time flow measurement (TTFM); ultrasound of endarterectomy site was used to evaluate problems if the flow was less than 100ml/min.

**Classification of Procedural Complications**

Bleeding complications were divided into three categories. In the first category was ICH due to hyperperfusion syndrome. In the second category were neck hematomas requiring evacuation. In the third category were all the patients who had prolonged hemostasis; this included all patients who had a comment on prolonged hemostasis during the surgery in the medical report, had large volumes of wound drainage postoperatively (over 500 mL) or an emergency department visit in which neck hematoma and/or bleeding were established.

**Supplemental table 1. Baseline demographics, comorbidities, medication, and outcomes in 143 patients with carotid stenosis with near-occlusion with and without full-collapse scheduled for carotid endarterectomy (CEA) in the Helsinki Carotid Endarterectomy Study 2 (HeCES2)**

|  | **ALL CS PATIENTS WITH NO,**  ***N* = 143** | **SUBTLE NO,**  **n= 124 (87 %)** | **NO WITH FULL COLLAPSE,**  ***n* = 19 (13%)** | ***p-*value** |
| --- | --- | --- | --- | --- |
| **Female gender (%)** | 43 (30 | 37 (30) | 6 (32) | 1.00 |
| **Age, mean±SD** | 69±9.1 | 70±8.46 | 65±11 | 0.310 |
| **Risk factors and comorbidities (%)** |  |  |  |  |
| Smoking | 60 (42) | 51 (41) | 9 (47) | 0.626 |
| Heavy alcohol consumption | 22 (15) | 18 (15) | 4 (21) | 0.495 |
| Hypercholesterolemia | 129 (90) | 113 (91) | 16 (84) | 0.400 |
| Arterial hypertension | 112 (78) | 101 (82) | 11 (58) | **0.033** |
| Diabetes mellitus | 54 (38) | 51 (41) | 3 (16) | **0.042** |
| Metabolic syndrome | 64 (45) | 61 (49) | 3 (16) | **0.006** |
| Coronary artery disease | 44 (31) | 42 (34) | 2 (11) | 0.059 |
| Peripheral arterial disease | 22 (15) | 19 (15) | 3 (16) | 1.000 |
| Atrial fibrillation | 17 (12) | 16 (13) | 1 (5.3) | 0.471 |
| Chronic kidney failure | 20 (14) | 19 (15) | 1 (5.3) | 0.474 |
| Previous carotid territory symptom | 21 (15) | 15 (12) | 6 (32) | **0.037** |
| **Index symptom (%)** |  |  |  |  |
| Ischemic stroke | 38 (27) | 30 (24) | 8 (42) | 0.160 |
| RAO | 6 (4.0) | 6 (4.8) | 0 | 1.000 |
| TIA | 27 (19) | 24 (19) | 3 (16) | 1.000 |
| AFX | 37 (26) | 33 (27) | 4 (21) | 0.781 |
| Asymptomatic and unclear origin of symptoms | 35 (25) | 31 (25) | 4 (21) | 1.000 |
| **Recanalization therapy** |  |  |  |  |
| IVT | 6 (4.2) | 6 (4.8) | 0 | 1.000 |
| EVT | 0 | 0 | 0 |  |
| **Medication use preoperatively (%)** |  |  |  |  |
| SAPT | 124 (87) | 108 (87) | 16 (84) | 0.719 |
| DAPT | 6 (4.2) | 5 (4.0) | 1 (5.3) | 0.582 |
| LMWH | 83 (58) | 74 (60) | 9 (47) | 0.329 |
| DM medication | 49 (34) | 47 (38) | 2 (11) | **0.020** |
| Antihypertensive medication | 110 (77) | 99 (80) | 11 (58) | **0.044** |
| Lipid-lowering medication ^e^ | 95 (66) | 83 (67) | 12 (63) | 0.796 |
| **Preoperative and procedural ~~perioperative~~ outcomes** |  |  |  | **Age-adjusted OR** |
| Recurrent iCVE | 19 (13.3) | 18 (14.5) | 1 (5.3) | 0.29 (0.04-2.4) |
| Recurrent stroke | 1 (0.7) | 1 (0.8) | 0 | 0.90 (0.9-1.0) |
| Recurrent TIA | 7 (4.9) | 7 (5.6) | 0 | 0.30 (0.04-2.5) |
| Recurrent AFX | 11 (7.7) | 10 (8.1) | 1 (5.3) | 0.51 (0.06-4.5) |
| **Procedural complications (%)** |  |  |  |  |
| Procedural iCVE | 7 (4.9) | 6 (4.8) | 1 (5.3) | 0.96 (0.88-1.05) |
| Procedural stroke | 7 (4.9) | 6 (4.8) | 1 (5.3) | 0.90 (0.09-8.2) |
| Procedural TIA/symptom worsening | 0 | 0 | 0 | ND |
| Procedural AMI/AP | 4 (2.8) | 3 (2.4) | 1 (5.3) | 2.9 (0.27-31) |
| Bleeding complication | 13 (9.1) | 3 (2.4) | 1 (5.3) | 0.58 (0.07-4.8) |
| Hyperperfusion syndrome | 10 (7.0) | 10 (8.1) | 0 | 0.92 (0.87-0.97) |
| Any stroke within 30 d | 8 (5.6) | 7 (5.6) | 1 (5.3) | 0.74 (0.08-6.9) |
| Any stroke or death within 30 d | 8 (5.6) | 7 (5.6) | 1 (5.3) | 0.74 (0.08-6.9) |

Data are presented as n (%) and mean ± standard deviation. The p value and OR is calculated between the s-NO and fc-NO groups.

CS = carotid stenosis, NO = near-occlusion, DM = diabetes mellitus, RAO = retinal artery occlusion, TIA = transient ischemic attack, AFX = amaurosis fugax, IVT= intravenous thrombolysis, EVT=endovascular treatment, AMI= acute myocardial infarction, AP= angina pectoris, iCVE= ischemic cerebrovascular event, SAPT= single antiplatelet therapy, DAPT=dual antiplatelet therapy, LMWH= low molecular wight heparin. *p* < 0.05 is considered statistically significant.

Refrences

1.	Nuotio K, Ijäs P, Heikkilä HM, et al. Morphology and histology of silent and symptom-causing atherosclerotic carotid plaques–Rationale and design of the Helsinki Carotid Endarterectomy Study 2 (the HeCES2). *Ann Med*. Published online 2018. doi:10.1080/07853890.2018.1494851
